# In Vitro Stability and Pharmacokinetic Study of Pedunculoside and Its Beta-CD Polymer Inclusion Complex

**DOI:** 10.3390/pharmaceutics16050591

**Published:** 2024-04-26

**Authors:** Liang Wu, Danfeng Li, Peijing Wang, Linling Dong, Wang Zhang, Jianjun Xu, Xiaoliang Jin

**Affiliations:** 1 School of Pharmacy, Nanjing University of Chinese Medicine, Nanjing 210023, China; 2 State Key Laboratory Cultivation Base for TCM Quality and Efficacy, Nanjing University of Chinese Medicine, Nanjing 210023, China; 3 School of Chemistry and Chemical Engineering, Yangzhou University, Yangzhou 225009, China; 4 Department of Applied Bioengineering, Graduate School of Convergence Science and Technology, Seoul National University, Seoul 08826, Republic of Korea; 5 Clinical Pharmacology Department, Changchun GeneScience Pharmaceutical Co., Ltd., Shanghai 200235, China

**Keywords:** pedunculoside, pedunculoside–βCDP, pharmacokinetic behavior, LC-MS/MS, method validation, intestinal metabolism

## Abstract

Pedunculoside, a triterpene saponin derived from various *Ilex* species, holds potential as a treatment for cardiovascular diseases. However, its clinical application is hindered by poor bioavailability, rapid elimination, and extensive intestinal metabolism to rotundic acid. To address these issues, a water-soluble inclusion complex of pedunculoside, namely, the beta-CD polymer inclusion complex of pedunculoside (pedunculoside–βCDP), was prepared in this study, and a comparative in vitro stability and pharmacokinetic behavior study was performed between pedunculoside and pedunculoside–βCDP. Both pedunculoside and pedunculoside–βCDP exhibited the highest stability in simulated gastric fluid and simulated intestinal fluid but were readily metabolized when co-incubated with *Bifidobacterium adolescentis* and *Bifidobacterium breve*. An LC-MS/MS analytical method for the simultaneous determination of pedunculoside and rotundic acid in rat plasma was successfully established, validated, and applied to investigate the pharmacokinetic behavior after rats were intravenously administered with pedunculoside or pedunculoside–βCDP. The results indicated that pedunculoside–βCDP could significantly improve the pharmacokinetic profile of pedunculoside by increasing plasma exposure, retarding elimination, and reducing intestinal metabolism. This study enhances our understanding of pedunculoside–βCDP’s metabolic fate and pharmacokinetic properties and potentially advances its further research, development, and clinical application.

## 1. Introduction

**Figure 1:**
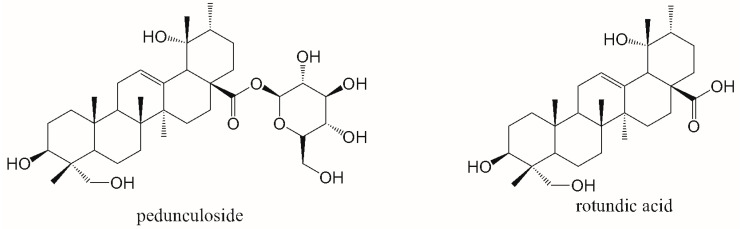
Chemical structures of pedunculoside and rotundic acid.

Despite the increasing use of synthetic drugs, bioactive components from herbal medicine remain a vibrant area of research due to their unique properties and potential synergy with existing therapies. Among these, pedunculoside [Figure 1], a triterpene saponin isolated from various *Ilex* species like *Ilex rotunda* Thunb and *Ilex pubescens* Hook. et Arn. [1,2,3], has emerged as a promising candidate for the treatment of cardiovascular diseases. Its diverse pharmacological effects, including attenuating ischemia–reperfusion injury [4,5], lowering blood lipids [6,7], and protecting against liver damage [8,9,10], suggest its potential for broad therapeutic applications. Notably, pedunculoside exhibits exceptional cardioprotective activity, prompting its exploration as a potential drug or prodrug candidate. Nevertheless, challenges such as its poor bioavailability, rapid elimination [with a short half-life time (t_1/2_)] [11,12], and extensive conversion into the less active metabolites (such as deglycosylated to generate its aglycon rotundic acid [Figure 1] by intestinal flora) [13,14,15], limit its clinical translation. Overcoming these pharmacokinetic hurdles could unlock the full therapeutic potential of pedunculoside and pave the way for its development as a novel, efficacious, and natural cardioprotective agent.

Cyclodextrin polymers (CDP) represent a class of sophisticated macromolecular structures derived from the assembly of cyclodextrin (CD) monomers. They combine the advantages of CD’s good complexation ability, high biocompatibility, and high water solubility, making them an effective drug carrier with excellent performance in drug delivery and controlled release. Furthermore, they have a wide range of applications in multiple fields. In previous works, our research group has built inclusion complexes of several herbal active ingredients based on CDPs, including pedunculoside, hypericin, ilexgenin A, and curcumin [16,17,18,19]. It has been confirmed that the inclusion complexation method can significantly increase the water solubility of drug molecules, improve their physicochemical properties, reduce toxicity, or reduce equivalent doses. Moreover, an increasing number of studies have reported that cyclodextrins can enhance the bioavailability of drugs [20,21]. Consequently, it is imperative to investigate whether cyclodextrins can similarly improve the bioavailability of pedunculoside, thereby facilitating its more widespread applications.

In this study, pedunculoside’s water-soluble inclusion complex, namely, its beta-CD polymer inclusion complex (pedunculoside–βCDP), was prepared. A comprehensive evaluation of its in vitro stability and pharmacokinetic profile was conducted in comparison to unmodified pedunculoside. The potential stability within the gastrointestinal environment was assessed using simulated gastric and intestinal fluids, along with specific gut microflora, including *Bifidobacterium adolescentis* and *Bifidobacterium breve*. Moreover, a precise LC-MS/MS analytical method was established and validated for the simultaneous determination of pedunculoside and rotundic acid in rat plasma. Plasma exposure and pharmacokinetic parameters of pedunculoside were calculated and estimated, and the generation of rotundic acid was also determined after rats were intravenously administered with pedunculoside or pedunculoside–βCDP. This is the first study to investigate the gastrointestinal tract stability, metabolism, and pharmacokinetic behavior of pedunculoside–βCDP. The findings will provide scientific evidence for its further research and development and facilitate its clinical application.

## 2. Materials and Methods

### 2.1. Chemicals and Materials

Chemical reference substances of pedunculoside and rotundic acid were purchased from Nanjing Liangwei Biological Technology Co., Ltd. (Nanjing, China); ilexsaponin A1 [used as an internal standard (IS) in the study] was purchased from Nanjing SenBeiJia Biological Technology Co., Ltd. (Nanjing, China). Simulated gastric fluid (SGF) and simulated intestinal fluid (SIF) were purchased from Phygene Biotechnology Co., Ltd. (Fuzhou, China). General anaerobic medium (GAM) broth was purchased from Beijing Solarbio Science and Technology Co., Ltd.; standard strains of *Bifidobacterium adolescentis* (CGMCC 1.2190) and *Bifidobacterium breve* (CGMCC 1.2213) were purchased from the China General Microbiological Culture Collection Center (CGMCC, Beijing, China). HPLC-grade methanol and acetonitrile were obtained from Merck (Darmstadt, Germany). Analytical-grade formic acid was purchased from Aladdin Industrial Corporation (Shanghai, China). A Milli-Q Gradient A10 ultrapure water system from Millipore (Milford, MA, USA) was used to prepare HPLC-grade water. All other chemicals were of reagent grade.

### 2.2. Chromatography and Mass Spectrometry Conditions

Chromatographic separation was performed using an Agilent Zorbax Eclipse Plus C18, 2.1 × 50 mm i.d., 3.5 μm column (Agilent Technologies, Santa Clara, CA, USA), on a Shimadzu LC-30A UFLC system (Shimadzu, Kyoto, Japan). The column oven temperature was set to 40 °C. A mobile phase consisting of 0.025% formic acid in water (A) and methanol (B) was delivered at a flow rate of 0.25 mL/min using the gradient program. The gradient program used was 20% (B) from 0 to 1.2 min, 20–65% (B) from 1.2 to 2.0 min, 65–90% (B) from 2.0 to 5.5 min, 90% (B) from 5.5 to 6.0 min, 90–20% (B) from 6.0 to 6.2 min, and 20% (B) from 6.2 to 7.8 min.

Detection and quantification were performed using an LC-MS/MS system (Triple Quad 4500/5500, Applied Biosystems, MDS SCIEX, Toronto, ON, Canada) that included Analyst software (Version 1.6.1) and MultiQuant software (Version 2.1). The mass operation parameters were as follows: ion source gas 1, 55 psi; ion source gas 2, 60 psi; curtain gas, 30 psi; collision gas, medium. A negative Turbo Spray ion source was used in the MS analysis. The temperature was set at 500 °C and the IonSpray voltage at −4500 V. Pedunculoside, rotundic acid, and ilexsaponin A1 (IS) were monitored via multiple reaction monitoring (MRM) mode. The precursor-to-product ion pairs for MRM transitions, declustering potential (DP), and collision energy (CE) were optimized at *m*/*z* 695.4 → 487.3, −120 V, and −36 V for pedunculoside; *m*/*z* 487.3 → 469.3, −130 V, and −42 V for rotundic acid; and *m*/*z* 663.4 → 501.3, −150 V, and −45 V for ilexsaponin A1, respectively.

### 2.3. Sample Preparation

The primary stock standard solutions of pedunculoside, rotundic acid, and ilexsaponin A1 (IS) were separately prepared in methanol at a concentration of 5 mg/mL and diluted with methanol as needed. All solutions were stored at 4 °C and brought to room temperature before use.

Pedunculoside–βCDP was prepared according to our previous study [16], and the molar ratio of pedunculoside to β-CDP was 1:2.

### 2.4. In Vitro Stability Study of Pedunculoside and Pedunculoside–βCDP in the Gastrointestinal Environment

First, 25 μL pedunculoside or pedunculoside–βCDP was added to 475 μL sterilized SGF or SIF (the final concentration of pedunculoside was approximately 1 μM) and incubated at 37 °C for 0 (negative control), 2, and 4 h. Simple, one-step protein precipitation with methanol was used to terminate the possible enzymatic and/or chemical reactions that may metabolize pedunculoside and extract pedunculoside from the SGF or SIF matrixes. Then, 200 µL of each sample was transferred and added to 4-fold volumes of methanol (800 μL) containing 10 ng/mL ilexsaponin A1 (IS). The mixture was vortexed for 3 min and centrifuged at 13,000 rpm for 10 min to separate the precipitated protein. The supernatant (200 µL) was then transferred and centrifuged at 13,000 rpm for another 10 min. Finally, 150 µL of the resulting supernatant was transferred, and a 2 µL aliquot of the supernatant from each sample was injected into LC-MS/MS (n = 3). Relative quantification was performed using an internal standard method to compare the relative content of pedunculoside among the groups.

Standard strains of *Bifidobacterium adolescentis* and *Bifidobacterium breve* were added to sterilized GAM broth and incubated at 37 °C under anaerobic conditions for about 2 days. Then, 2 μL pedunculoside or pedunculoside–βCDP was mixed with 198 μL each kind of precultured strain (the final concentration of pedunculoside was approximately 1 μM) and anaerobically incubated at 37 °C and 120 rpm for 0 (negative control) and 2 days. To confirm the metabolic capability of the bacteria, sterilized GAM broth without any strain was used as a blank control. To terminate each potential metabolic reaction and extract both pedunculoside and rotundic acid from the broth matrix, 4-fold volumes of methanol (800 μL) containing 10 ng/mL ilexsaponin A1 (IS) were added. The mixture was vortexed for 3 min and centrifuged at 13,000 rpm for 10 min twice. A 2 µL aliquot of the supernatant from each sample was injected into LC-MS/MS (n = 3). Relative quantification was performed using an internal standard method to compare the relative content of pedunculoside and rotundic acid among the groups.

### 2.5. Simultaneous Determination of Pedunculoside and Rotundic Acid in Rat Plasma—Sample Pretreatment

Simple protein precipitation with methanol was used to extract both pedunculoside and rotundic acid from rat plasma. Then, 50 µL of each plasma sample was added to 4-fold volumes of methanol (200 μL) containing 10 ng/mL ilexsaponin A1 (IS). The mixture was vortexed for 3 min and centrifuged at 13,000 rpm for 10 min to separate the precipitated protein. The supernatant (200 µL) was transferred and centrifuged at 13,000 rpm for another 10 min. Finally, 150 µL of the resulting supernatant was transferred, and a 2 µL aliquot of the supernatant from each sample was injected into LC-MS/MS.

Working solutions of pedunculoside and rotundic acid with concentrations in the range of 50–20,000 ng/mL and 100–20,000 ng/mL were obtained by serially diluting the stock solution with methanol. The calibration standards were prepared by spiking the series of working solutions (2.5 μL) into drug-free rat plasma (47.5 μL) to yield pedunculoside concentrations of 2.5, 5, 10, 25, 50, 100, 250, 500, 1000 ng/mL and rotundic acid concentrations of 5, 10, 25, 50, 100, 250, 500, 1000 ng/mL and then treated by protein precipitation as stated above. For each calibration curve, a double blank sample was prepared from 50 μL of drug-free rat plasma, and protein precipitation was performed using 200 μL of blank methanol (without IS).

Quality control (QC) samples were prepared in the same way as calibration standards. The concentrations of pedunculoside in the QC samples were 5, 50, and 500 ng/mL, and the concentrations of rotundic acid were 10, 50, and 500 ng/mL. The samples were then treated by protein precipitation as stated above.

### 2.6. Simultaneous Determination of Pedunculoside and Rotundic Acid in Rat Plasma—Method Validation

Selectivity: Blank plasma samples from six different rats were treated with blank methanol (without IS) to ensure no interference would disturb the detection and quantification of both the analytes and IS. Each blank sample was analyzed and compared with the samples at low concentrations in the calibration curves.

Calibration curve: The calibration curves were constructed by plotting the ratio of the peak area of pedunculoside or rotundic acid to that of IS (y) vs. the nominal concentration of calibration standards (x) and fitted to linear regression (y = ax + b) using 1/x as the weighting factor. Six replications of the calibration curves were constructed. To evaluate the linearity and assess the lower limit of quantification (LLOQ), each calibration equation and the corresponding correlation coefficient (r^2^) were recorded, and the concentrations of pedunculoside and rotundic acid at each nominal concentration were also calculated by the regression parameters from the calibration curves.

Precision and accuracy: The precision and accuracy were evaluated by analyzing the QC samples at three (low, medium, and high) concentrations. To determine the intra- and inter-day variability, six replicates of each QC sample were prepared and analyzed on three consecutive validation days. The calibration curve constructed on the same testing day was used for the quantification of each QC sample.

Recovery and matrix effect: Recovery of the pretreatment procedure for both pedunculoside and rotundic acid from rat plasma was estimated at three different concentrations of QC samples by comparing the responses obtained from blank plasma samples spiked with the analytes before protein precipitation with those from the post-pretreated samples at equivalent concentrations (n = 6). The matrix effect was measured by comparing the responses of pedunculoside and rotundic acid spiked in plasma matrix to ultrapure water at three different QC levels following the same protein precipitation process using 4-fold volumes of methanol (n = 6). The ratios give the percentage recovery and matrix effect.

Stability: QC samples at three concentrations were also used. Autosampler stability was assessed by analyzing the processed QC samples being stored in the autosampler at 4 °C for 24 h. To investigate room temperature stability, freeze and thaw stability, and freeze storage stability, QC samples were first prepared to certain concentrations and pretreated after being stored at room temperature for 4 h, undergoing three freeze–thaw cycles (−20 °C to room temperature as one cycle), and then stored at −20 °C for one week. All the testing QC samples were determined using the freshly prepared calibration curves.

### 2.7. Pharmacokinetic Behavior Study of Pedunculoside after Pedunculoside or Pedunculoside–βCDP Was Intravenously Injected to Rats

The experimental procedures were approved by the Animal Experimental Ethical Committee of Nanjing University of Chinese Medicine (202305A016) and conducted in compliance with the ethical principles of animal use and care. All possible measures were taken to diminish pain, suffering, and distress in animals while also striving to limit the quantity of animals utilized.

A total of 12 male Sprague–Dawley rats weighing 220 ± 30 g were purchased from Shanghai Bikai Keyi Biotechnology Co., Ltd. (Shanghai, China) and housed in an environmentally controlled room (temperature: 20 ± 2 °C, humidity: 60 ± 5%, 12 h dark–light cycle). Food and water were given ad libitum. After 1-week acclimatation, rats were fasted for at least 8 h with free access to water prior to the experiment and equally divided into two groups at random (n = 6). In group one, pedunculoside was carefully weighed and dissolved in 5% DMSO and 95% sterilized normal saline solution at a concentration of 1 mg/mL, and a dose of 5 mg/kg pedunculoside was intravenously administered to each rat by rapid injection via the tail vein. In group two, pedunculoside–CDP was directly dissolved in sterilized normal saline solution, with the concentration of pedunculoside (already in the form of pedunculoside–βCDP) also at 1 mg/mL, and a dose of 22.44 mg/kg pedunculoside–βCDP (containing pedunculoside in the water-soluble beta-CD polymer inclusion complex equal to the dose of 5 mg/kg pedunculoside) was also intravenously administered to each rat. About 150 μL of blood samples were collected in heparinized polyethylene tubes before administration and at 5, 15, 30, 45, 60, 90, 120, 180, 240, 360, and 480 min after administration from the fossa orbitalis vein of the rats. After centrifugation at 4000 rpm for 10 min, 50 μL of plasma samples were obtained and stored at −20 °C. After being brought to room temperature, rat plasma samples were processed as described in the “2.5. Simultaneous Determination of Pedunculoside and Rotundic Acid in Rat Plasma—Sample Pretreatment” section.

### 2.8. Data Process

All peaks were integrated using MultiQuant software (Version 2.1).

To investigate the stability of pedunculoside or pedunculoside–βCDP, relative quantification through MRM mode was performed by recording the peak area ratios of pedunculoside or rotundic acid to ilexsaponin A1 (IS). The peak area ratios in different incubating conditions on the vertical Y-axis against pedunculoside and pedunculoside–βCDP groups on the horizontal X-axis were plotted using the GraphPad Prism 5.0 software (GraphPad Software, Inc.; San Diego, CA, USA).

To determine the concentration of pedunculoside and rotundic acid in rat plasma, peak area ratios of pedunculoside or rotundic acid to ilexsaponin A1 (IS) in each sample were recorded and substituted into the freshly prepared calibration curves to obtain the absolute plasma concentrations of pedunculoside and rotundic acid. During the determination process of the real samples, a series of QC samples at three concentrations were evenly distributed across the sample test sequence (>5%) to ensure the reliability of the quantitative results.

Pharmacokinetic parameters were calculated using a non-compartmental method using the Phoenix WinNonlin 8.4 software (CERTARA, Pharsight, Princeton, NJ, USA). Data are reported as the mean ± standard deviation (SD). Statistical assessments were conducted using two-tailed Student’s *t*-tests. Statistical significance was established at levels of * *p* < 0.05, ** *p* < 0.01, and *** *p* < 0.001. Analysis of the statistical data was conducted with the GraphPad Prism software (Version 5.01).

## 3. Results

### 3.1. In Vitro Stability Study of Pedunculoside and Pedunculoside–βCDP in the Gastrointestinal Environment

**Figure 2:**
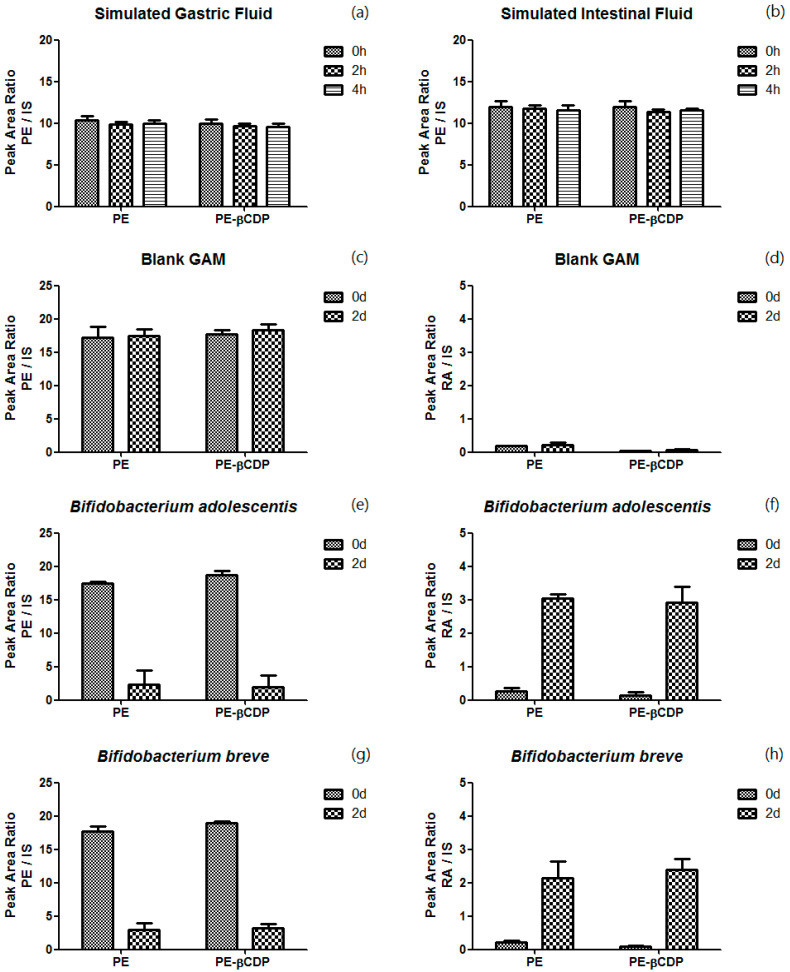
In vitro stability study of pedunculoside and pedunculoside–CDP in the gastrointestinal environment of (**a**,**b**) simulated gastric and intestinal fluids, (**c**,**d**) sterilized GAM broth, (**e**,**f**) *Bifidobacterium adolescentis*, and (**g**,**h**) *Bifidobacterium breve* (n = 3) (PE: pedunculoside, PE–βCDP: pedunculoside–βCDP, RA: rotundic acid).

Stability of pedunculoside in a gastrointestinal environment was assessed through co-incubation in simulated gastric and intestinal fluids and with the specific gut microflora *Bifidobacterium adolescentis* and *Bifidobacterium breve*, which have been proven to metabolize pedunculoside in our previous study [14]. The relative quantification of pedunculoside or its metabolite, rotundic acid, was characterized using the peak area ratio of pedunculoside or rotundic acid to the internal standard. The experimental findings revealed that both pedunculoside and pedunculoside–βCDP exhibited the highest stability in SGF and SIF [Figure 2a,b], as indicated by the consistent peak area ratio of pedunculoside to IS observed when incubated in SGF or SIF for up to 4 h at 1 μM. In contrast, a significant reduction in the peak area ratio of pedunculoside to IS was observed when pedunculoside and pedunculoside–βCDP were incubated with *Bifidobacterium adolescentis* and *Bifidobacterium breve* for 48 h [Figure 2e,g], which was further corroborated by comparison with sterilized GAM broth (the blank control) [Figure 2c]. In the meantime, rotundic acid (the aglycon of pedunculoside) was found to be generated, as the peak area ratio of rotundic acid to IS increased obviously [Figure 2d,f,h].

### 3.2. Simultaneous Determination of Pedunculoside and Rotundic Acid in Rat Plasma—Method Validation

**Figure 3:**
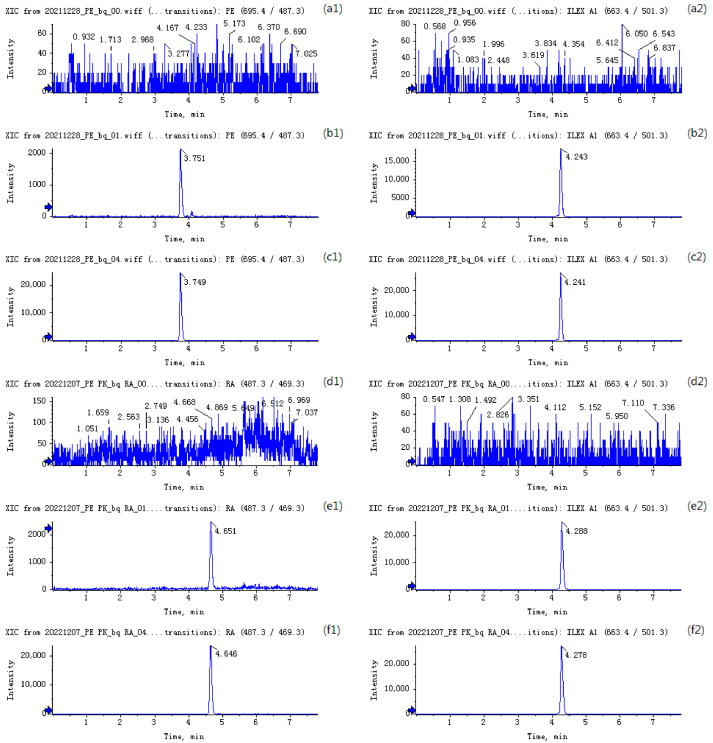
Representative chromatograms for pedunculoside and IS in (**a1**,**a2**) blank rat plasma, (**b1**,**b2**) blank rat plasma spiked with 2.5 ng/mL pedunculoside, and (**c1**,**c2**) blank rat plasma spiked with 20 ng/mL pedunculoside. Representative chromatograms for rotundic acid and IS in (**d1**,**d2**) blank rat plasma, (**e1**,**e2**) blank rat plasma spiked with 5 ng/mL rotundic acid, and (**f1**,**f2**) blank rat plasma spiked with 50 ng/mL rotundic acid.

Selectivity: Within the experimental conditions established, it was observed that all the analytes were well separated, with a fine peak shape and stable retention times. The retention times of pedunculoside, rotundic acid, and IS were 3.8, 4.6, and 4.2 min, respectively, with no significant interference observed from the blank rat plasma matrix. Representative chromatograms of the analytes obtained from the blank rat plasma sample and the blank rat plasma sample added with pedunculoside or rotundic acid (at LLOQ and a medium concentration) are shown in Figure 3.

Calibration curve: The linearity was investigated with the preparation and injection of the calibration curve (2.5–1000 ng/mL for pedunculoside and 5–1000 ng/mL for rotundic acid) on six different batches. Both pedunculoside and rotundic acid exhibited satisfactory calibration linearity, with correlation coefficient values greater than 0.99 (Table S1). Consequently, LLOQ for pedunculoside and rotundic acid was regarded as 2.5 and 5 ng/mL. The accuracy of each concentration used in the calibration curves calculated by the corresponding calibration equations was also acceptable (Table S1).

Precision and accuracy: The intra-day and inter-day precision and accuracy of the analytical method are shown in Table 1. In this assay, the intra-day and inter-day precision variation at each QC level was within 0.55–7.62% for pedunculoside and within 3.35–9.24% for rotundic acid; the RE of accuracy was within ±8.95% for pedunculoside and within ±10.78% for rotundic acid. These results indicate that our analytical method is reproducible with satisfactory precision and accuracy.

Recovery and matrix effect: As shown in Table 2, the recovery rates in rat plasma at three QC levels were 98.57% ± 9.03%, 104.25% ± 2.35%, and 103.56% ± 5.01% for pedunculoside and 111.10% ± 8.55%, 98.25% ± 2.71%, and 100.15% ± 4.12% for rotundic acid. With regard to the matrix effect, the calculated values at three QC levels were 107.70% ± 7.89%, 102.53% ± 6.28%, and 96.42% ± 4.61% for pedunculoside and 107.70% ± 9.82%, 104.54% ± 3.24%, and 108.70% ± 4.55% for rotundic acid. Such results are acceptable and reproducible. No significant matrix interference was observed, indicating that no co-eluting substance influenced the ionization of the analytes, and no signal suppression or enhancement existed under the analytical condition.

Stability: The stability results of pedunculoside and rotundic acid at three QC levels are also summarized in Table 2. For pedunculoside, the RSD values at three QC levels ranged from 1.96% to 8.70%, and RE values were within ±7.70%; for rotundic acid, the RSD values at three QC levels were between 2.91% and 11.12%, and RE values were within ±8.09%. The results demonstrated that both pedunculoside and rotundic acid offered satisfactory stability under autosampler conditions at 4 °C for 24 h, followed by room temperature for 4 h, undergoing three freeze–thaw cycles, and being stored at −20 °C for one week.

### 3.3. Pharmacokinetic Behavior Study of Pedunculoside after Pedunculoside or Pedunculoside–βCDP Was Intravenously Injected to Rats

**Figure 4:**
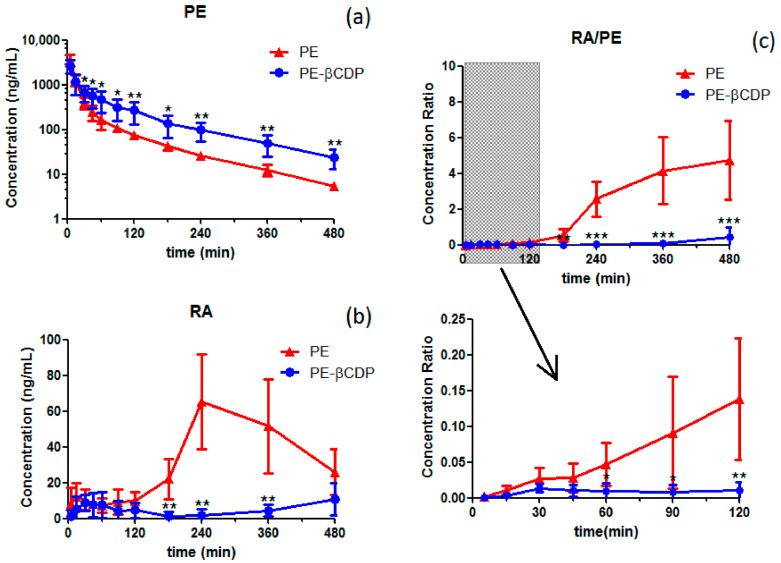
Mean plasma concentration–time curves of (**a**) pedunculoside, (**b**) rotundic acid, and (**c**) pedunculoside vs. rotundic acid in rats (n = 6) (PE: pedunculoside, PE–βCDP: pedunculoside–βCDP, RA: rotundic acid). Differences were considered significant at * *p* < 0.05, ** *p* < 0.01, and *** *p* < 0.001.

The mean plasma concentration–time curves of pedunculoside after intravenous administration of pedunculoside (5 mg/kg) or pedunculoside–βCDP (22.44 mg/kg, containing pedunculoside equal to 5 mg/kg) are shown in Figure 4a, and the main pharmacokinetic parameters are summarized in Table 3. It can be observed from Figure 4a that following administration of the same dose of pedunculoside, the mean plasma concentration in the pedunculoside–βCDP group was significantly higher than that in the pedunculoside group.

The results indicated that pedunculoside was rapidly eliminated, with a half-life time (t_1/2_) of 99.968 ± 18.284 min and a mean residence time (MRT) of 48.598 ± 8.362 min. After being administered for 480 min, plasma concentration was only left at 5.488 ± 1.037 ng/mL. When administered in the form of pedunculoside–βCDP, it was observed that t_1/2_ extended to 129.641 ± 44.217 min, MRT extended to 112.004 ± 12.419 min, and plasma concentration at 480 min (C_480min_) increased to 24.113 ± 11.415 ng/mL. In addition, AUC increased from 81914.111 ± 12666.038 to 121065.16 ± 48490.659 min·ng/mL, and clearance (CL) decreased from 62.194 ± 9.033 to 49.214 ± 24.335 mL/min/kg. Among all the pharmacokinetic parameters, it was found that C_480min_ and MRT showed an extremely significant difference (*p* < 0.01) when pedunculoside–βCDP was used.

Additionally, the primary metabolite, rotundic acid (the aglycon of pedunculoside), was also detected and peaked after 240 min administration of pedunculoside (Figure 4b). When administered in the form of pedunculosid–βCDP, hardly any rotundic acid was observed to be generated (Figure 4b). The concentration ratio of rotundic acid to pedunculoside at different times was also estimated (Figure 4c) and compared. It was observed that when pedunculoside was administered, the ratio increased with time, especially after 120 min; however, it did not change much when pedunculoside–βCDP was given.

## 4. Discussion

### 4.1. In Vitro Stability Study of Pedunculoside and Pedunculoside–βCDP in the Gastrointestinal Environment

Pedunculoside is a triterpenoid saponin. SGF and SIF were used to test whether it was stable in an acidic or alkaline medium during its absorption or metabolism. According to the results, pedunculoside is not likely to be degraded within the normal pH range in vivo in the form of both dissociative pedunculoside and its water-soluble beta-CD polymer inclusion complex.

On the other hand, prior research from our group had identified that *Bifidobacterium adolescentis* and *Bifidobacterium breve* possess the capacity to metabolize pedunculoside to generate its aglycon rotundic acid [14], prompting the selection of these bacterial strains to assess whether the inclusion complex pedunculoside–βCDP would exhibit enhanced stability against intestinal microflora. According to the results, however, pedunculoside–βCDP did not show any superiority compared with pedunculoside in the in vitro stability study, so gastrointestinal administration (especially if orally taken) of pedunculoside or pedunculoside–βCDP should be avoided as this would certainly lead to severe first-pass effects.

### 4.2. Simultaneous Determination of Pedunculoside and Rotundic Acid in Rat Plasma—Method Validation

Rotundic acid is the aglycon of pedunculoside and is also the main metabolite of pedunculoside, proven to be generated through pedunculoside deglycosylation through intestinal flora [13,14,15]. Therefore, when an analytical method is established, both pedunculoside and rotundic acid should be taken into consideration to obtain a comprehensive understanding of pedunculoside pharmacokinetics. However, this was not designed or deliberated on in previous works [11,12,22].

Overall, the LC-MS/MS analytical method we established is accurate, sensitive, and reliable, with all the methodological indexes tested (selectivity, calibration curve, precision and accuracy, recovery, matrix effect, and stability) meeting the requirements of biological sample analysis. The method could realize the simultaneous determination of pedunculoside and rotundic acid in rat plasma within 8 min, monitor and estimate the pharmacokinetic behavior of pedunculoside, and reveal its potential biotransformation to rotundic acid.

### 4.3. Pharmacokinetic Behavior Study of Pedunculoside after Pedunculoside or Pedunculoside–βCDP Was Intravenously Injected to Rats

Cyclodextrin complexes have been widely prepared and applied to various bioactive ingredients in the pharmaceutical field, including anti-infective drugs, antihypertensive drugs, antiepileptic drugs, anti-tumor drugs, antihyperglycemic drugs, and common non-steroidal anti-inflammatory drugs [23,24,25]. In previous studies, researchers were mainly concerned with improving water solubility and relative physicochemical properties, as well as obtaining superior therapeutic effects with less toxicity. A comprehensive pharmacokinetic study of cyclodextrin complexes would be beneficial to reveal the potential pharmacodynamic mechanism and provide useful information to facilitate their clinical use. According to the current study, by creating an inclusion complex of altrenogest with hydroxypropyl-β-cyclodextrin, its oral bioavailability was improved [20]. By complexation with hydroxypropyl-γ-cyclodextrin, griseofulvin showed better pharmacokinetic parameters in dogs [21]. Although a water-soluble inclusion complex of pedunculoside with the polymer β-cyclodextrin (pedunculoside–βCDP) was successfully developed and characterized several years ago [16], this is the first study to investigate its in vivo pharmacokinetic behavior.

According to the results of the in vitro stability study, to circumvent the intricate effects of intestinal bacterial metabolism on pedunculoside absorption, intravenous administration was used in the pharmacokinetic study. The pharmacokinetic parameters obtained by us of pedunculoside administered intravenously to rats were similar to those of previous studies [11,12]. We also demonstrated that pedunculoside was eliminated quickly in vivo; thus, its pharmacodynamic effect would not last long. While its water-soluble inclusion complex, pedunculoside–βCDP, was tested, it was found in the PK concentration time curve that the plasma concentration of pedunculoside in the pedunculoside–βCDP group was significantly higher than that in the pedunculoside group, while the concentration of its main metabolite (rotundic acid) and the concentration ratio of rotundic acid to pedunculoside were significantly lower. Moreover, the pharmacokinetic parameters of C_480min_ and MRT showed an extremely significant difference (*p* < 0.01) compared with the normal pedunculoside group. For the other pharmacokinetic parameters, although a statistically significant difference was not determined due to the great individual differences among the animals, the plasma exposure of pedunculoside was substantially improved (AUC increased), and the elimination of pedunculoside was also delayed (t_1/2_ extended and CL decreased). Overall, it was sufficient enough to illustrate that pedunculoside–βCDP exhibited better pharmacokinetic behavior than pedunculoside alone.

During the excretory process, pedunculoside can be metabolized by the intestinal bacteria to generate rotundic acid, and rotundic acid can then enter the blood circulation through intestinal absorption. This was the reason why rotundic acid was detected with time, especially after 180 min. By contrast, when pedunculoside–CDP was administered, the intestinal metabolism of pedunculoside was greatly reduced, and the concentration level of rotundic acid was almost negligible. Comparing the concentration ratio of rotundic acid to pedunculoside at different times, it is believed that the metabolism process of pedunculoside could be significantly avoided using the water-soluble inclusion complex pedunculoside–βCDP.

## 5. Conclusions

In this study, we prepared pedunculoside–βCDP and performed a systematic comparative metabolism and pharmacokinetic study between pedunculoside and pedunculoside–βCDP. Our findings revealed that both pedunculoside and pedunculoside–βCDP were most stable in simulated gastric fluid and simulated intestinal fluid and were easily metabolized when co-incubated with *Bifidobacterium adolescentis* and *Bifidobacterium breve*. No significant differences in in vitro stability were observed between pedunculoside–βCDP and pedunculoside. To investigate their pharmacokinetic profile, including the potential intestinal metabolism, an LC-MS/MS analytical method for the simultaneous determination of pedunculoside and rotundic acid in rat plasma was successfully established and validated. After rats were intravenously administered with pedunculoside or pedunculoside–βCDP, pharmacokinetic parameters of pedunculoside were calculated, and plasma exposure to rotundic acid was also determined. The data revealed that pedunculoside–βCDP could significantly improve the pharmacokinetic behavior of pedunculoside by increasing the plasma exposure (increased plasma concentration and AUC), slowing down the elimination (prolonged t_1/2_ and MRT and decreased clearance), and reducing the intestinal metabolism (decreasing the generation of rotundic acid). Taken together, these data contribute to a better understanding of pedunculoside–βCDP metabolism and pharmacokinetics, promote its further research and development, and provide scientific evidence for its clinical application.

## Figures and Tables

**Table 1:** Precision and accuracy of pedunculoside and rotundic acid in rat plasma.

Spiked Concentration (ng/mL)		Intra-Day (D1, n = 6)		Intra-Day (D2, n = 6)		Intra-Day (D3, n = 6)		Inter-Day (n = 3)	
		Measured Concentration (ng/mL)	RSD	Measured Concentration (ng/mL)	RSD	Measured Concentration (ng/mL)	RSD	Measured Concentration (ng/mL)	RSD
	5	4.95 ± 0.38	7.71%	5.37 ± 0.36	6.72%	5.26 ± 0.27	5.12%	5.19 ± 0.22	4.26%
Pedunculoside	50	53.15 ± 3.11	5.85%	54.47 ± 2.32	4.27%	54.35 ± 1.27	2.34%	53.99 ± 0.73	1.35%
	500	516.83 ± 17.33	3.35%	519.20 ± 27.02	5.20%	522.34 ± 16.89	3.23%	519.46 ± 2.76	0.53%
	10	9.29 ± 0.73	7.86%	11.08 ± 0.50	4.48%	10.28 ± 0.92	8.99%	10.22 ± 0.89	8.75%
Rotundic acid	100	110.78 ± 4.51	4.07%	104.09 ± 6.66	6.40%	107.54 ± 3.53	3.28%	107.47 ± 3.35	3.11%
	500	475.81 ± 23.51	4.94%	542.05 ± 26.07	4.81%	486.70 ± 25.20	5.18%	501.52 ± 35.52	7.08%

**Table 2:** Matrix effect, recovery and stability of pedunculoside and rotundic acid in rat plasma.

Spiked Concentration (ng/mL)			Matrix Effect (%)	Recovery (%)	Stability (%)			
					24 h in Autosampler	4 h at Room Temperature	3 Freeze–Thaw Cycles	Frozen Storage for One Week
		Mean	107.70	98.57	96.97	92.30	99.66	95.57
	5	SD	7.89	9.03	7.80	4.15	8.70	3.04
		RSD	7.32	9.16	8.05	4.49	8.73	3.18
		Mean	102.53	104.25	106.76	106.22	100.97	98.01
Pedunculoside	50	SD	6.28	2.35	3.62	4.30	4.60	2.81
		RSD	6.13	2.25	3.40	4.05	4.55	2.86
		Mean	96.42	103.56	103.17	96.34	100.80	97.30
	500	SD	4.61	5.01	1.96	3.07	4.37	2.49
		RSD	4.78	4.84	1.90	3.18	4.34	2.56
		Mean	107.70	111.10	108.09	102.09	99.09	100.17
	10	SD	9.82	8.55	6.95	7.24	11.12	2.91
		RSD	9.12	7.70	6.43	7.10	11.22	2.90
		Mean	104.54	98.25	105.08	99.85	98.29	92.85
Rotundic acid	50	SD	3.24	2.71	9.29	5.34	6.80	3.41
		RSD	3.10	2.76	8.85	5.35	6.92	3.67
		Mean	108.70	100.15	96.09	95.03	94.84	103.39
	500	SD	4.55	4.12	3.13	4.42	5.97	5.69
		RSD	4.19	4.12	3.26	4.65	6.29	5.50

**Table 3:** Main pharmacokinetic parameters of pedunculoside after intravenous injection of pedunculoside and pedunculoside–βCDP in rats (n = 6, mean ± SD).

Parameter	Parameter Given from the Software	Unit	Pedunculoside	Pedunculoside–βCDP
k	Lambda_z	1/min	0.007126 ± 0.0012735	0.0057647 ± 0.0015335
t_1/2_	HL_Lambda_z	min	99.968 ± 18.284	129.641 ± 44.217
C_480min_	Clast	ng/mL	5.488 ± 1.037	24.113 ± 11.415 **
AUC_0→t_	AUClast	min·ng/mL	81107.642 ± 12835.326	116598.701 ± 47591.249
AUC_0→∞_	AUCINF_obs	min·ng/mL	81914.111 ± 12666.038	121065.16 ± 48490.659
AUC_t→∞_/AUC_0→∞_	AUC_%Extrap_obs	%	1.031 ± 0.492	3.993 ± 2.011
CL	Cl_obs	ml/min/kg	62.194 ± 9.033	49.214 ± 24.335
MRT	MRTINF_obs	min	48.598 ± 8.362	112.004 ± 12.419 ***

**: *p* < 0.01, ***: *p* < 0.001.

## Data Availability

The data sets used and/or analyzed during this study are available from the corresponding author upon request.

## Supplementary Materials

The following supporting information can be downloaded at https://www.mdpi.com/article/10.3390/pharmaceutics16050591/s1, Table S1: Detailed information of the calibration curves of pedunculoside and rotundic acid in rat plasma; Figure S1: Plasma concentration–time curves for pedunculoside in rats after intravenous administration of pedunculoside; Figure S2: Plasma concentration–time curves for pedunculoside in rats after intravenous administration of pedunculoside–βCDP.

## References

[1] Yi F., Zhao X.-L., Peng Y., Xiao P.-G. (2016). Genus *Ilex* L.: Phytochemistry, Ethnopharmacology, and Pharmacology. Chin. Herb. Med..

[2] Yang B., Li H., Ruan Q.-F., Xue Y.-Y., Cao D., Zhou X.-H., Jiang S.-Q., Yi T., Jin J., Zhao Z.-X. (2018). A facile and selective approach to the qualitative and quantitative analysis of triterpenoids and phenylpropanoids by UPLC/Q-TOF-MS/MS for the quality control of *Ilex rotunda*. J. Pharm. Biomed. Anal..

[3] Jiang S.-Q., Cui H., Wu P., Liu Z.-Q., Zhao Z.-X. (2019). Botany, traditional uses, phytochemistry, pharmacology and toxicology of *Ilex pubescens* Hook et Arn. J. Ethnopharmacol..

[4] Liu J.-X., Zhang Y.-H. (2017). Study on the effect of the presence of pedunculoside on myocardial ischemia in rats. Smart Healthc..

[5] Yang A.-P., Zhang Q.-C., Song Y., Yang M., Wang J.-Y., Wang C., Hu L.-H., Wang X.-C. (2021). Study on protective effect of pedunculoside on acute myocardial ischemia/reperfusion injury in rats. Chin. Pharmacol. Bull..

[6] Rong Y., Shen Y.-J., Wang J., Zhang Y.-Q., Fan Y., Sun Y., Sun W.-D. (2016). The effects of pedunculoside on the liver injury in mice and the gastrocnemius damage in rat. J. Yangzhou Univ..

[7] Liu C., Shen Y.-J., Tu Q.-B., Zhao Y.-R., Guo H., Wang J., Zhang L., Shi H.-W., Sun Y. (2018). Pedunculoside, a novel triterpene saponin extracted from *Ilex rotunda*, ameliorates high-fat diet induced hyperlipidemia in rats. Biomed. Pharmacother..

[8] Ma X., Chen G., Wang J., Xu J., Zhao F., Hu M., Xu Z., Yang B., Guo J., Sun S. (2019). Pedunculoside attenuates pathological phenotypes of fibroblast-like synoviocytes and protects against collagen-induced arthritis. Scand. J. Rheumatol..

[9] Chen X., Cheng B., Song N., Wu Y.-L., Li Y.-W. (2019). Effect of pedunculoside on MiR-29a/TET3 and STAT3 in mice with colitis-associated colorectal cancer (CAC). Pharmacol. Clin. Chin. Materia Med..

[10] Liu K.-J., Li G.-F., Guo W.-J., Zhang J. (2020). The protective effect and mechanism of pedunculoside on DSS (dextran sulfate sodium) induced ulcerative colitis in mice. Int. Immunopharmacol..

[11] Zhao W.-O., Pang L., Xu D.-H., Zhang N. (2015). LC-MS/MS determination and pharmacokinetic study of pedunculoside in rat plasma after oral administration of pedunculoside and *Ilex rotunda* extract. Molecules.

[12] Yang A.-P., Dong J.-J., Zhao H.-M., Zhang Q.-C., Zhu X.-Y., Gao L.-N., Ding N., Li C.-H., Peng R., Lu T.-L. (2021). Quantification and pharmacokinetics study of pedunculoside in rats by using UPLC-MS/MS. Curr. Pharm. Anal..

[13] Cao D., Fan Z., Zhu J.-P., Yang B., Zhou L., Jin J., Zhao Z.-X. (2016). Effect of rat intestinal bacteria on metabolism of pedunculoside in vitro. China Pharm..

[14] Wu L., Kang A., Shan C.-X., Chai C., Zhou Z., Lin Y.-J., Bian Y.-Y. (2019). LC-Q-TOF/MS-oriented systemic metabolism study of pedunculoside with in vitro and in vivo biotransformation. J. Pharm. Biomed. Anal..

[15] Yang B., Li H., Ruan Q.-F., Xuan S.-X., Chen X.-J., Cui H., Liu Z.-Q., Jin J., Zhao Z.-X. (2019). Effects of gut microbiota and ingredient-ingredient interaction on the pharmacokinetic properties of rotundic acid and pedunculoside. Planta Med..

[16] Liu C., Zhang W., Yang H., Sun W.-D., Gong X.-D., Zhao J.-X., Sun Y., Diao G.-W. (2014). A water-soluble inclusion complex of pedunculoside with the polymer β-cyclodextrin: A novel anti-inflammation agent with low toxicity. PLoS ONE.

[17] Zhang W., Gong X.-D., Cai Y., Zhang C.-L., Yu X., Fan J., Diao G.-W. (2013). Investigation of water-soluble inclusion complex of hypericin with β-cyclodextrin polymer. Carbohydr. Polym..

[18] Liu C., Zhang W., Wang Q., Sun Y., Diao G.-W. (2013). The water-soluble inclusion complex of ilexgenin A with β-cyclodextrin polymer-a novel lipid-lowering drug candidate. Org. Biomol. Chem..

[19] Zhang W., Xiao P., Lin L.-W., Guo F., Wang Q.-Y., Piao Y.-Z., Diao G.-W. (2022). Study of a water-soluble supramolecular complex of curcumin and β-cyclodextrin polymer with electrochemical property and potential anti-cancer activity. Chin. Chem. Lett..

[20] Chen W.-J., Zheng X.-W., Lao W.-X., Wang H.-X., Chen S.-F., Liu C.-Y., Chen Z.-S., Bai Y.-S., Zhang H., Zhan X.-S. (2024). Enhancement of the solubility and oral bioavailability of altrenogest through complexation with hydroxypropyl-β-cyclodextrin. Eur. J. Pharm. Sci..

[21] Ding Y.-L., Cui W.-T., Zhang Z.-Y., Ma Y.-Z., Ding C., Lin Y.-K., Xu Z. (2023). Solubility and Pharmacokinetic Profile Improvement of Griseofulvin through Supercritical Carbon Dioxide-Assisted Complexation with HP-γ-Cyclodextrin. Molecules.

[22] Zhao W.-Z., Ju W.-Z., Zang Y.-X., Sun B.-T., Tan H.-S. (2016). Pharmacokinetics of pedunculosid in rat. Chin. J. New Drugs Clin. Rem..

[23] Liu H., Guo S.-L., Wei S.-J., Liu J.-Y., Tian B.-R. (2024). Pharmacokinetics and pharmacodynamics of cyclodextrin-based oral drug delivery formulations for disease therapy. Carbohydr. Polym..

[24] Sarabia-Vallejo Á., Caja MD M., Olives A.-I., Martín M.-A., Menéndez J.-C. (2023). Cyclodextrin Inclusion Complexes for Improved Drug Bioavailability and Activity: Synthetic and Analytical Aspects. Pharmaceutics.

[25] Zhang W., Zheng Z.-Q., Lin L.-W., Zhang X., Bae M., Lee J., Xie J., Diao G.-W., Im H.-J., Piao Y.-Z. (2023). Ultrafast Synthesis of Graphene-Embedded Cyclodextrin-Metal-Organic Framework for Supramolecular Selective Absorbency and Supercapacitor Performance. Adv. Sci..

