# Supplementary material for: In Vitro Stability and Pharmacokinetic Study of Pedunculoside and Its Beta-CD Polymer Inclusion Complex
Source: Pharmaceutics. 2024 Apr 26;16(5):591. doi: 10.3390/pharmaceutics16050591 (PMC11125186; doi:10.3390/pharmaceutics16050591)
Supplement: Supplementary file 1 [file pharmaceutics-16-00591-s001.zip › pharmaceutics-2940751-supplementary.pdf]

In vitro Stability and Pharmacokinetic Study of Pedunculoside and its Beta-CD Polymer Inclusion Complex

Liang Wu [1,2,*], Danfeng Li [1], Peijing Wang [1], Linling Dong [1], Wang Zhang [3,4], Jianjun Xu [1], and Xiaoliang Jin [5,*]

[1] School of Pharmacy, Nanjing University of Chinese Medicine, Nanjing 210023, China

[2] State Key Laboratory Cultivation Base for TCM Quality and Efficacy, Nanjing University of Chinese Medicine, Nanjing 210023, China

[3] School of Chemistry and Chemical Engineering, Yangzhou University, Yangzhou 225009, China

[4] Department of Applied Bioengineering, Graduate School of Convergence Science and Technology, Seoul National University, Seoul 08826, Republic of Korea

[5] Clinical Pharmacology Department, Changchun GeneScience Pharmaceutical Co., Ltd. Shanghai 200235, China

* Corresponding author:

Dr. Liang Wu,

State Key Laboratory Cultivation Base for TCM Quality and Efficacy, School of Pharmacy, Nanjing University of Chinese Medicine, 138 Xianlin Road, Nanjing 210023, China; E-mail: wuliang@njucm.edu.cn

Dr. Xiaoliang Jin,

Clinical Pharmacology Department, Clinical Research and Development Center, Changchun GeneScience Pharmaceutical Co., Ltd., 88 Hongcao Road, Shanghai 200235, China; E-mail: jinxiaoliang@genscigroup.com

Table S1. Detail information of the calibration curves of pedunculoside and rotundic acid in rat plasma

pedunculoside	calibration curve	r^2	Accuracy (%) of the Spiked Concentration (ng/mL)								
			2.5	5	10	20	50	100	200	500	1000
1	y = 0.04632 x + 0.05326	0.99777	105.14	95.07	85.49	104.97	108.54	100.65	96.32	106.73	97.09
2	y = 0.04389 x + 0.03438	0.99709	87.73	101.95	99.77	103.06	109.93	95.77	97.19	108.05	96.55
3	y = 0.04689 x + 0.02684	0.99816	103.66	104.10	99.16	94.91	109.77	94.15	90.86	101.79	101.59
4	y = 0.03754 x + 0.01463	0.99783	98.45	106.05	100.49	94.17	100.06	98.83	97.25	107.57	97.13
5	y = 0.03710 x + 0.01711	0.99867	99.31	106.58	95.85	101.89	99.11	100.27	92.90	104.68	99.41
6	y = 0.03812 x + 0.01741	0.99720	90.52	97.88	100.24	101.21	111.13	99.13	95.33	107.70	96.85

rotundic acid	calibration curve	r^2	Accuracy (%) of the Spiked Concentration (ng/mL)							
			5	10	20	50	100	200	500	1000
1	y = 0.01289 x + 0.03038	0.99918	82.83	98.80	104.81	110.66	104.28	98.16	101.95	98.50
2	y = 0.01021 x + 0.02882	0.99938	85.51	106.41	103.43	108.83	97.44	99.93	97.40	101.06
3	y = 0.01252 x + 0.02143	0.99730	81.73	96.34	110.84	111.14	109.62	91.46	96.79	102.08
4	y = 0.01312 x - 0.00318	0.99938	107.16	108.08	97.53	92.47	96.81	96.04	100.54	101.36
5	y = 0.01167 x + 0.01430	0.99912	89.50	94.91	106.15	100.13	107.74	104.49	98.07	99.01
6	y = 0.01374 x + 0.01433	0.99850	89.30	104.26	104.37	104.52	103.05	91.39	103.18	99.93

Subject=1

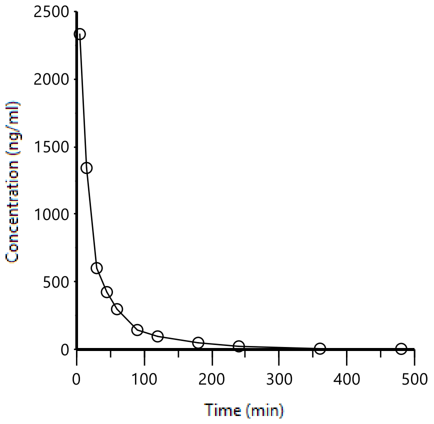

Subject=1

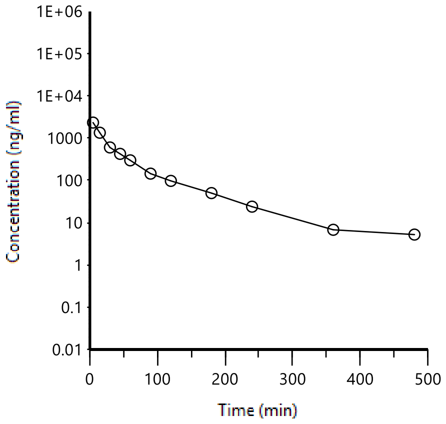

Subject=2

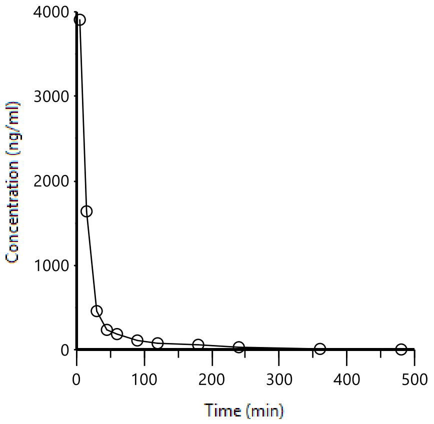

Subject=2

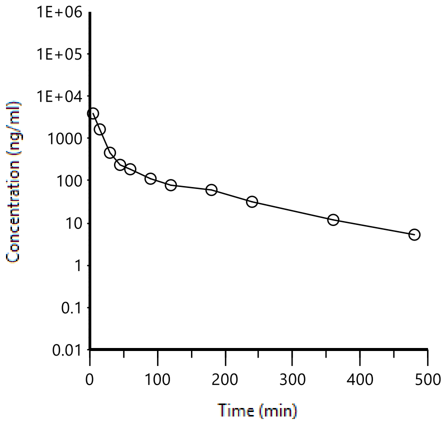

Subject=3

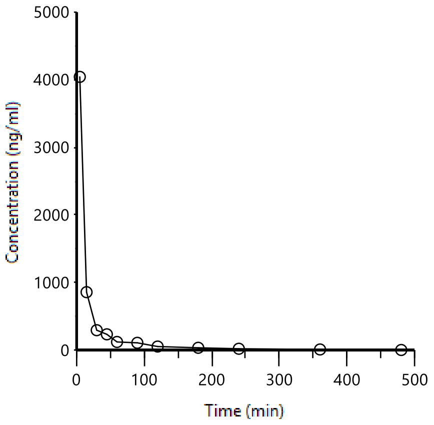

Subject=3

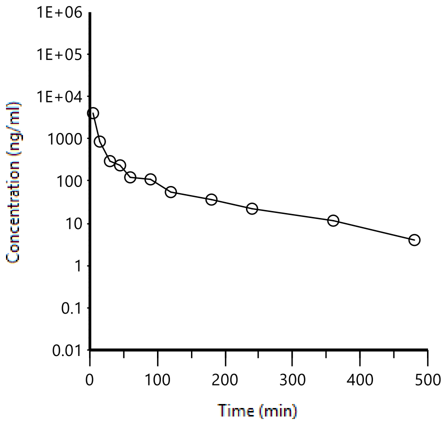

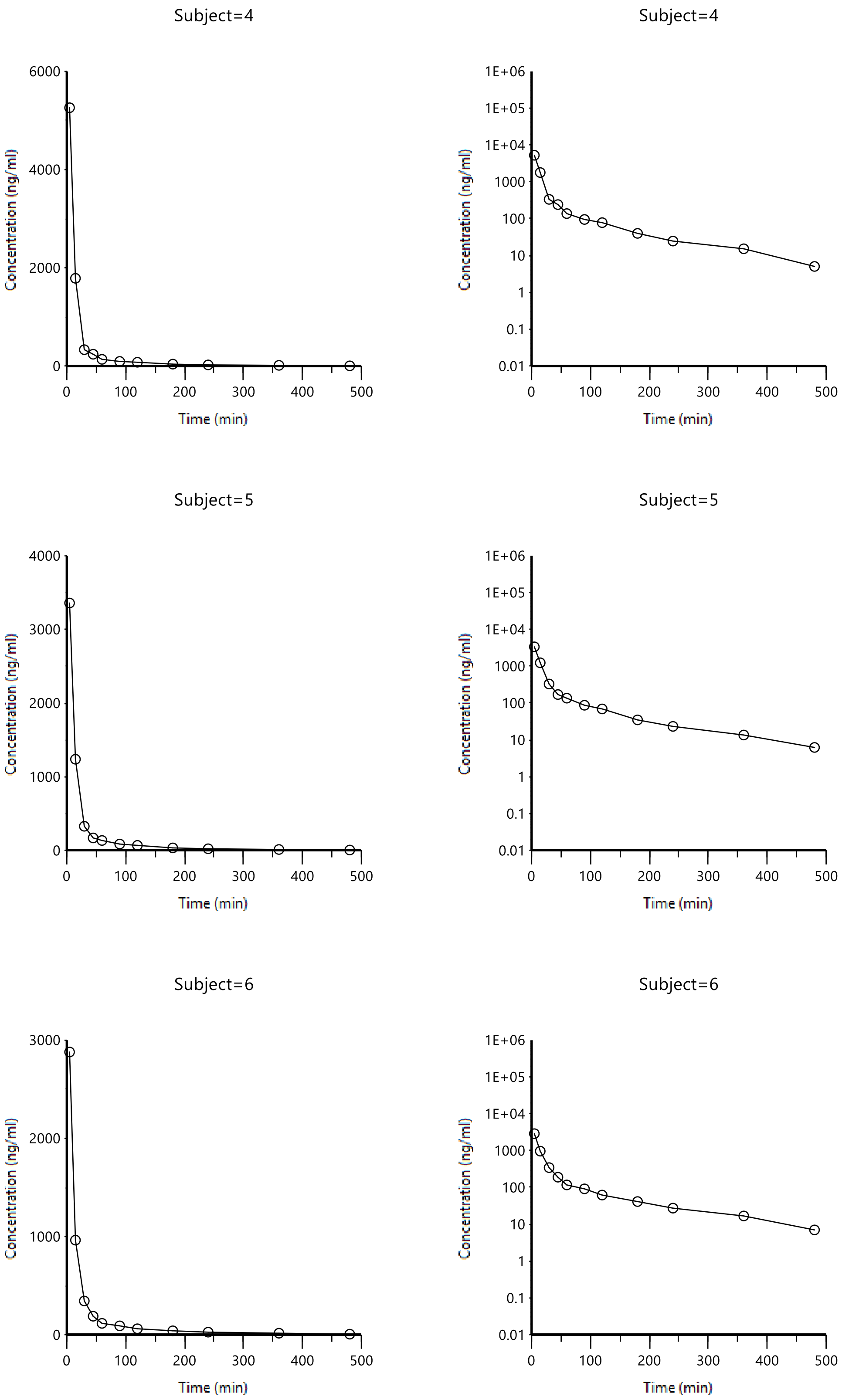

Figure S1. Plasma concentration-time curves for pedunculoside in rats after intravenous administration of pedunculoside

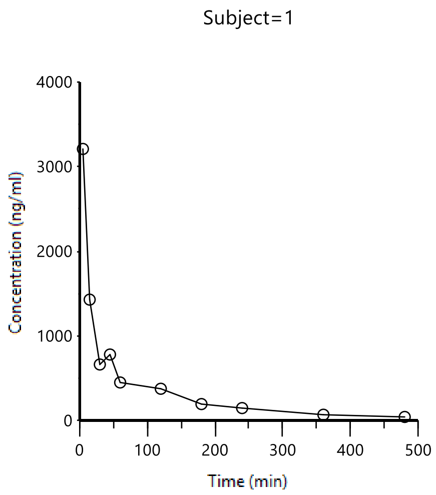
Subject=1
4000
3000
2000
1000
0
Concentration (ng/ml)
0
100
200
300
400
500
Time (min)

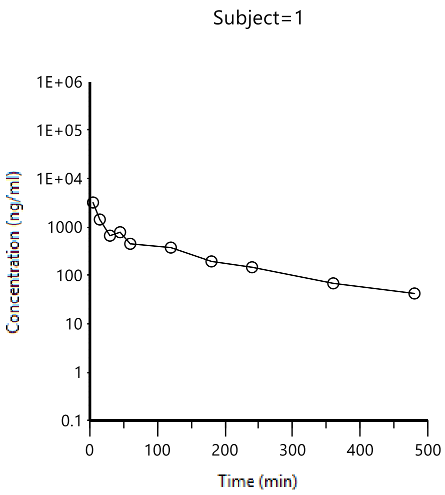
Subject=1
1E+06
1E+05
1E+04
1000
100
10
1
0.1
Concentration (ng/ml)
0
100
200
300
400
500
Time (min)

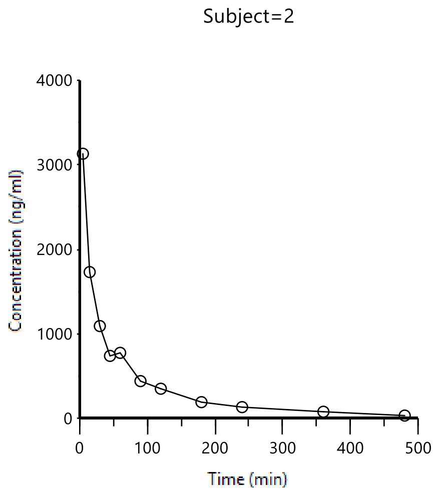
Subject=2
4000
3000
2000
1000
0
Concentration (ng/ml)
0
100
200
300
400
500
Time (min)

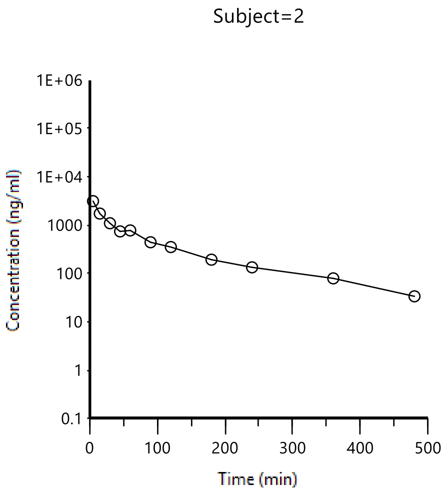
Subject=2
1E+06
1E+05
1E+04
1000
100
10
1
0.1
Concentration (ng/ml)
0
100
200
300
400
500
Time (min)

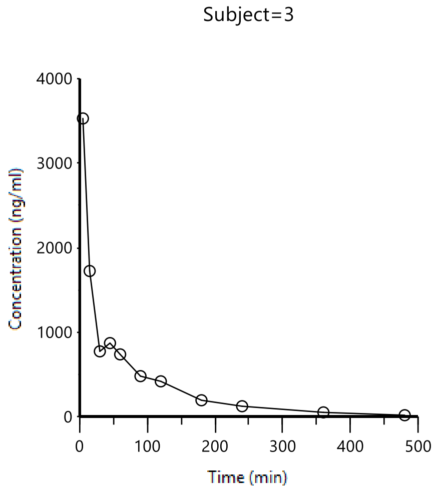
Subject=3
4000
3000
2000
1000
0
Concentration (ng/ml)
0
100
200
300
400
500
Time (min)

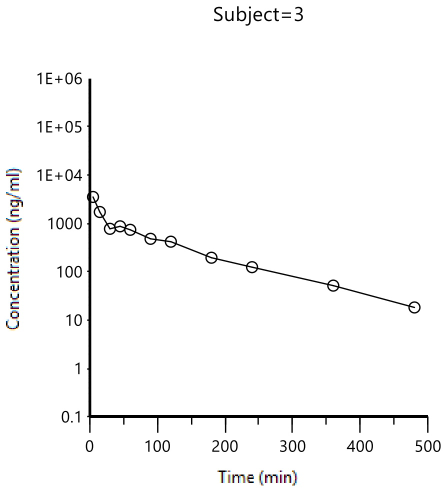
Subject=3
1E+06
1E+05
1E+04
1000
100
10
1
0.1
Concentration (ng/ml)
0
100
200
300
400
500
Time (min)

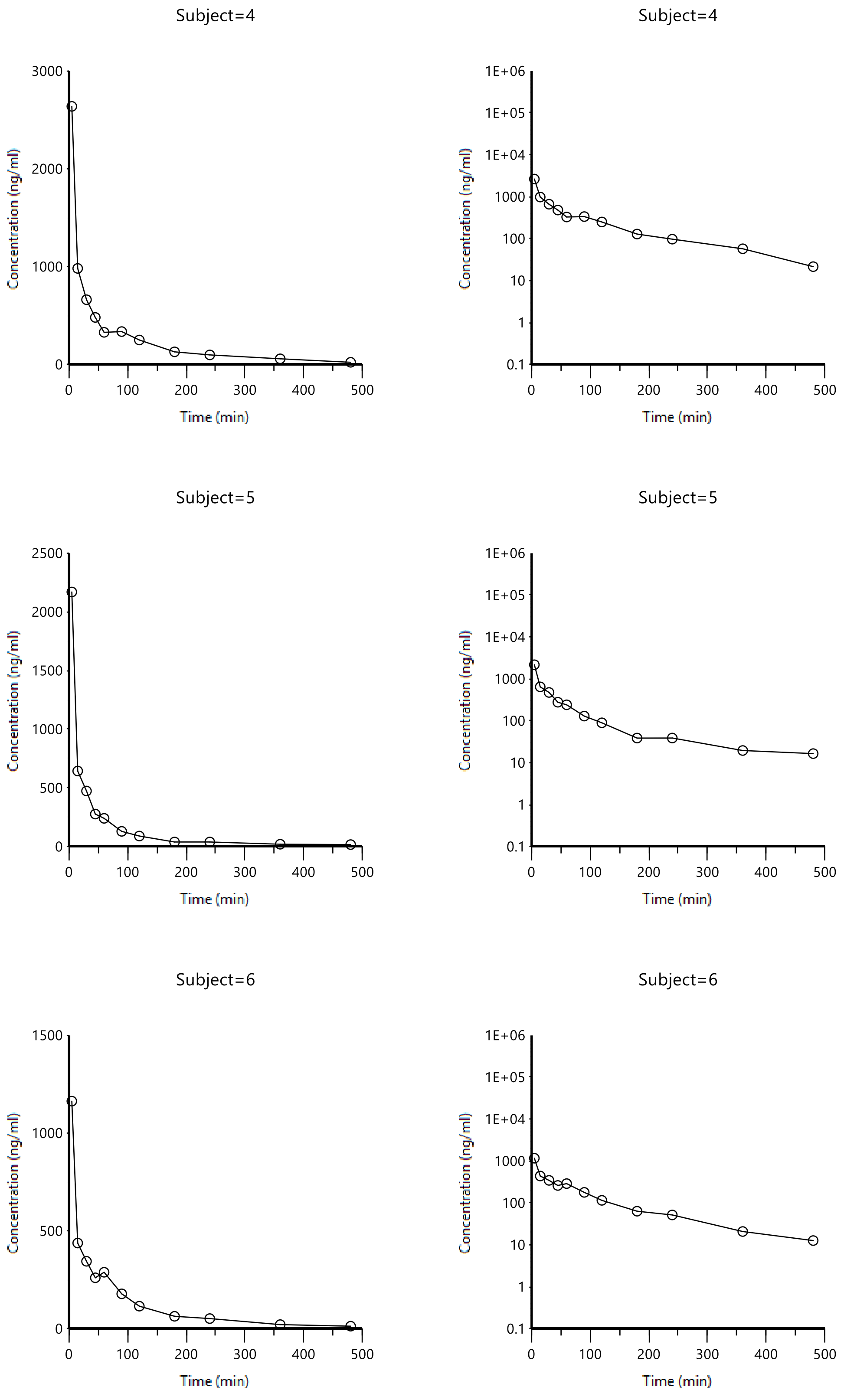

Figure S2. Plasma concentration-time curves for pedunculoside in rats after intravenous administration of pedunculoside-βCDP
